# Breast cancer incidence in food- *vs* non-food-producing areas in Norway: possible beneficial effects of World War II

**DOI:** 10.1038/sj.bjc.6600084

**Published:** 2002-02-01

**Authors:** T E Robsahm, S Tretli

**Affiliations:** KREFTREGISTERET, Institute of Population-Based Cancer Research, Montebello, 0310 Oslo, Norway; Department of Community Medicine and General Practice, The Norwegian University of Science and Technology, Trondheim, Norway

**Keywords:** breast cancer, residence, childhood/adolescence, energy restriction

## Abstract

It has been suggested that World War II influenced breast cancer risk among Norwegian women by affecting adolescent growth. Diet changed substantially during the war, and the reduction in energy intake was assumed to be larger in non-food- producing than in food-producing municipalities. In the present study, we have looked at the influence of residential history in areas with and without food production on the incidence of breast cancer in a population-based cohort study consisting of 597 906 women aged between 30 and 64 years. The study included 7311 cases of breast cancer, diagnosed between 1964 and 1992. The risk estimates were calculated using a Poisson regression model. The results suggest that residential history may influence the risk of breast cancer, where the suggested advantageous effect of World War II seems to be larger in non-food-producing than in food-producing areas. Breast cancer incidence was observed to decline for the post-war cohorts, which is discussed in relation to diet.

*British Journal of Cancer* (2002) **86**, 362–366. DOI: 10.1038/sj/bjc/6600084 www.bjcancer.com

© 2002 The Cancer Research Campaign

## 

It has been suggested that breast cancer incidence is particularly sensitive to influences early in life. Associations have been observed between birth weight (Michels *et al*, 1996; Ziegler, 1998) and other perinatal factors (Ekbom *et al*, 1992; Potischman and Troisi, 1999) and breast cancer incidence. In addition, the maturing of the breast between menarche and the first full-term pregnancy is thought to be important in breast cancer development (Moolgavkar *et al*, 1980; Russo *et al*, 1982; Tretli and Gaard, 1996). Effects on breast cancer has been demonstrated among women who were exposed to radiation during adolescence – from the atom bomb (Tokunaga *et al*, 1994) and in treatment for Hodgkin's disease (Bhatia *et al*, 1996). An ecological study found that women whose puberty was during World War II (when food was restricted) had a lower breast cancer risk than expected (Tretli and Gaard, 1996). Other ecological studies have also underlined the possible importance of food intake (Doll and Peto, 1981; De Waard and Trichopoulos, 1988; Potishman *et al*, 1998). Furthermore, the observed positive association between body height and breast cancer is attributed to dietary conditions in early life (Tretli, 1989; Vatten *et al*, 1992; Van der Brandt *et al*, 1997; Lund Nilsen and Vatten, 2001).

In Norway, there has for a long time been a contrast between the incidence of breast cancer in urban and that in rural areas. During World War II the Norwegian population experienced food restriction, as well as changes in the foods consumed. These changes were more pronounced in towns and in areas that did not produce food (Statistics Norway, 1946; Strøm, 1948).

The present study aimed, therefore, to examine the influence of residential history, in areas with and without food production, on breast cancer incidence in relation to the dietary effects of World War II.

## MATERIALS AND METHODS

Statistics Norway conducted a national population census in 1960, which consisted of all inhabitants living in Norway at this time. Based on this census, all inhabitants were assigned an 11-digit personal identification number, which was put into use in 1964; this gave rise to the opportunity to register vital statistics about the Norwegian population. Since 1964 the Central Population Registry has compulsorily registered all migration to or from Norway, and between municipalities within the country. All cancer cases diagnosed since 1953 have been compulsorily reported to the Cancer Registry of Norway.

From this census, the following information was used for each person: place of birth, year of birth, vital status, residence data, reproductive data and data about education and occupation. Cancer information was obtained by linkage to the Cancer Registry of Norway using the personal number of every citizen.

The median age of a first full-term pregnancy for birth cohorts during the period 1935–1953 was 23 (Brunborg and Kravdal, 1986). Hence, the time before age 23 represent childhood and adolescence, after age 23 the time represents adult life.

The municipalities were classified into areas with (Food) and without food (Non-food) production, according to the main income sources for the municipalities in 1968 (Statistics Norway, 1974). The Food areas consist of municipalities with a main income from agriculture, hunting and fishing. The Non-food areas are municipalities with income mainly from secondary- and service industries. A woman's residential category was set to Non-food if she lived for more than 70% of her lifetime before the age of 23 and ⩾70% of her life after the age of 23 in Non-food municipalities. The corresponding definition applies to the Food category.

The study is based on a total of 2 255 814 women registered as living in Norway since 1960. Women born between 1926 and 1960, aged between 30 and 64 years during the observation period 1964–1992 (*n*=740 531), and who fulfilled the criteria of a residence category, were included. This resulted in 597 906 women, including 7311 women with breast cancer. The closing date of follow-up was the date of diagnosis, death, migration from Norway or a cut-off date of 31 December 1992.

Number of children and age at first birth were recorded in the census for each woman. In the analyses, the following categories of age at first birth were used: nulliparous women and women with the first child born when aged less than 20, between the ages of 20–24, 25–29 or aged 30 years or more.

Educational levels were categorized based on the number of years of education: low (1–9 years), medium (10–12 years) and high (⩾13 years) educational level. Based on the information from the census, according to International standard classification of occupations 1958 (International Labour Office, 1962), three levels of occupational physical activity were used: occupations with high, medium and low level of physical activity.

### Statistical methods

To investigate the relationship between residential history and the incidence of breast cancer, a model of age, period and cohort was applied, based on the Poisson distribution. Stratified analyzes by residence category were performed. Estimation and testing were carried out using the EPICURE statistical program (Preston *et al*, 1993). The population was divided into 5-year birth cohorts (1926–1930 through to 1956–1960) and 5-year age groups (30–34 through to 60–64). As birth cohort and age gave the period of diagnosis, the periods overlapped (synthetic periods), covering the years 1960–1992. An acceptable goodness of fit, appraised by the deviance, was achieved when only age and birth cohorts were included in the model. Thus, the period of diagnosis was left out of the final models.

Risk estimates were calculated as the incidence per 100 000 person-years and relative risks (RR), with 95% confidence intervals (95% CI). The effects of potentially confounding variables, available in this study, were evaluated by including age at first birth, and level of education and occupational activity in the analyses.

To illustrate the pattern of breast cancer incidence in Norway during the twentieth century and for comparison with results obtained previously (Tretli and Gaard, 1996) the incidence were estimated by age at diagnosis (30–34 through to 55–59) and birth cohorts since 1901 (1901–1905 through to 1955–1960). The period of follow-up was between 1953 and 1992.

## RESULTS

Table 1

**Table 1:**
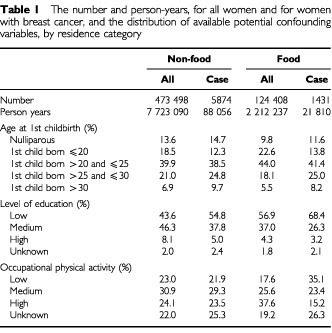
The number and person-years, for all women and for women with breast cancer, and the distribution of available potential confounding variables, by residence category

presents the number and person-years, for all women and for women with breast cancer, separately; the distribution of available potential confounding variables is presented by residence category. The women in Non-food areas are more frequently nulliparous and have a delayed child-bearing pattern compared with women in Food areas. In Food areas, women tend to be less educated, but have a higher level of occupational physical activity compared with women in Non-food areas. These potentially confounding factors have changed by birth cohort unfavourably with regard to breast cancer risk, particularly in Non-food areas (not shown). If these potentially confounding factors were solely of importance, this would imply that any expected difference in breast cancer incidence between areas should increase in post-war cohorts.

Table 2

**Table 2:**
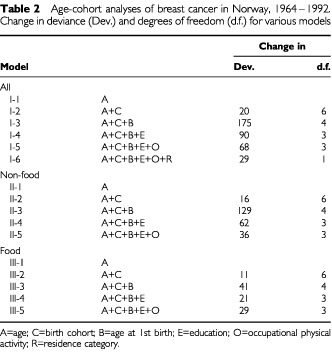
Age-cohort analyses of breast cancer in Norway, 1964–1992. Change in deviance (Dev.) and degrees of freedom (d.f.) for various models

describes how the different models fit the data. All the variables included showed significant improvement in the model, except for the period of diagnosis (not shown). In particular, age at first birth contributed strongly. Model I-6 (Table 2) demonstrated a significant improvement when adding residence area (R) to the model, even when potentially confounding variables were taken into account. Interaction terms between residence and each of the variables C-O (Table 2) were added one by one to model I-6, but none of them contributed significantly (not shown).

Figure 1

**Figure 1:**
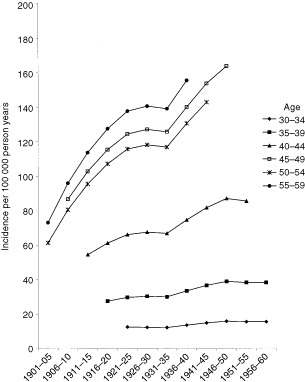
The estimated incidence of breast cancer in Norwegian women by age at diagnosis and birth cohort (Model I-2).

shows an updated version of an age-cohort-model previously described and discussed by Tretli and Gaard (1996). It suggests that the special nutritional conditions during World War II influenced, beneficially, the breast cancer risk in later life in women who were between thelarche and age at first birth during the war. Figure 1, covering birth-cohorts between 1901 and 1960, was not adjusted for residence, child-bearing patterns, educational levels or occupational physical activity. Such data were available from the 1926 birth cohort and Figure 2

**Figure 2:**
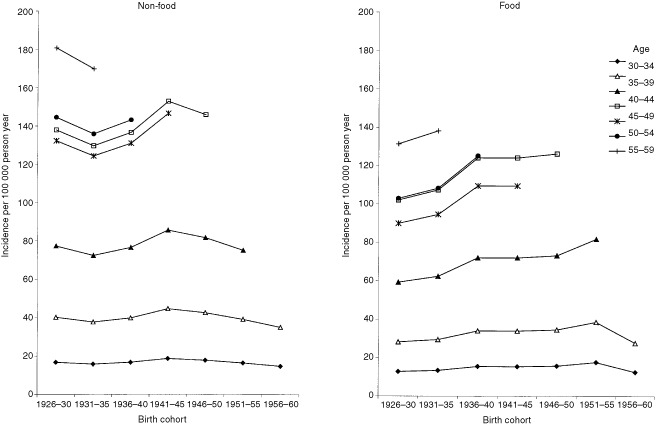
The estimated incidence of breast cancer by age at diagnosis and birth cohort, for Food and Non-food areas, models II-5 and III-5 (adjusted for age at first birth and level of education and occupational physical activity).

presents separately the adjusted estimated incidence curves for Non-food and Food areas, based on models II-5 and III-5 (Table 2). The suggested benefit of World War II on breast cancer risk seems to be expressed more in Non-food than in Food areas.

It is notable, from Figure 2, that breast cancer risk decreased by birth cohort after the war in Non-food areas; a similar tendency is seen in Food areas some years later.

Table 3

**Table 3:**
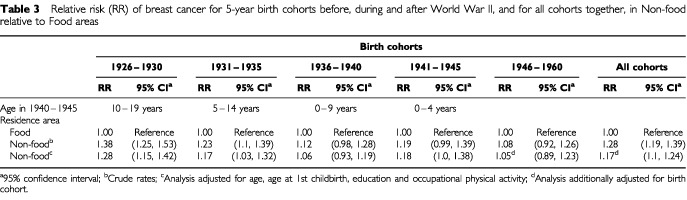
Relative risk (RR) of breast cancer for 5-year birth cohorts before, during and after World War II, and for all cohorts together, in Non-food relative to Food areas

shows the higher risk of breast cancer in Non-food compared with Food areas for the birth cohorts 1926–1935. Furthermore, it shows a diminishing risk difference between the two areas for the younger birth cohorts. Only a slight difference can be observed for the post-war cohorts, even after adjusting for variables that were supposed to strengthen the difference between the areas.

## DISCUSSION

In Norway, there has been an increasing incidence of breast cancer over time, however, the incidence seems to have levelled off for women who were teenagers during the World War II (Figure 1). The main factor, discussed earlier, was the reduced food availability and energy intake during World War II, which were sufficient to influence the height and weight of children (Brundtland *et al*, 1980). The army of occupation imposed restrictions, which meant a substantial reduction in energy intake and consumption of fat, meat and milk, and an increase in consumption of fish, cereals, fresh vegetables and potatoes. Although the restrictions on the food supply applied all over the country, the effects of the rationing were more noticeable in Non-food areas (Strøm, 1948). When residence area is included in the analyses, it is in an attempt to give an indication of food consumed. The suggested effect of World War II on the risk of breast cancer would therefore be expected to be more pronounced in Non-food areas, which seems to have been observed in the present study.

Of course, the residential areas are different in many aspects. The child-bearing pattern has been different, as well as the educational levels and occupational possibilities. From previous studies, it is known that these factors may also influence breast cancer risk (Chie *et al*, 2000; Verloop *et al*, 2000), and the differences should point towards a higher incidence in Non-food areas. Furthermore, these factors have changed unfavourably by increasing birth cohorts, particularly in Non-food areas.

The level of physical activity increased during the war (Ustvedt, 1961). A higher level of occupational physical activity was observed in Food areas compared with Non-food areas. Information about physical activity in leisure time was not available from the census, but there is no support for any difference in this between the two area categories. All available factors were included in the analyses, although the adjustments did not influence the result that the difference in incidence between areas with and those without food-production diminished by birth cohort (Table 3).

The present results may support the hypothesis that energy deprivation at certain ages is a determinant of breast cancer. A reduced stature as a result of energy restriction early in life may explain the reduced incidence of cancer in Non-food relative to Food areas. Energy restriction during the growth years is associated with reduced adult stature and a reduced number of breast tissue cells, which affects the risk of breast cancer (De Waard and Trichopoulos, 1988). Lund Nilsen and Vatten (2001) found supporting results in a recent Norwegian cohort study. A positive association was observed between adult height and breast cancer, for women born during a period of nutritional diversity (World War II). They attributed this observation to the role of early nutrition in breast cancer aetiology. In the Netherlands Dirx *et al* (1999) observed no substantial change of breast cancer risk in women who experienced the war famine of 1944–1945. However, they suggested that the birth cohorts included in the study had passed by a believed sensitive period at the time of restriction. Furthermore, the authors indicated that energy restriction before puberty influences the risk of breast cancer, which also corresponds to the results presented in Table 3 (birth cohorts 1936–1940).

The age at menarche was not substantially changed during the war (Liestøl, 1982); hence the biological explanation of the benefit of energy-restriction on residents in Non-food areas is probably not the result of a delay in the onset of menses.

The tendency for a decline in incidence in post-war cohorts corresponds to the observation by Tretli and Gaard (1996), although it is more pronounced because younger birth-cohorts have been included.

During World War II, Strøm and Jensen (1951) observed a drop in death from myocardial infarction in line with a drop in fat consumption. After the war the mortality from infarction increased up to the 1970s, since when it has decreased, initially in urban areas. This observed behaviour is of interest in view of the trends in the present study, which suggest that energy restriction during World War II influences later breast cancer risk and, furthermore, the incidence was observed to decline in post-war cohorts, primarily in Non-food areas. It may therefore be factors related to food habits that are common for these two diseases.

In conclusion, the results indicate that residential history may influence the risk of breast cancer. The suggestion that World War II had a beneficial influence on breast cancer risk seems to be evident more in Non-food than in Food areas, which supports the influence of nutrition during the growth years on breast cancer risk. Furthermore, breast cancer incidence appears to decline in post-war cohorts, and later on in Food areas. In discussion about future studies of diet and breast cancer, energy restriction and patterns of exposure to myocardial infarction risk factors could be taken into account.
